# Pertactin-deficient *Bordetella pertussis* isolates: evidence of increased circulation in Europe, 1998 to 2015

**DOI:** 10.2807/1560-7917.ES.2019.24.7.1700832

**Published:** 2019-02-14

**Authors:** Alex-Mikael Barkoff, Jussi Mertsola, Denis Pierard, Tine Dalby, Silje Vermedal Hoegh, Sophie Guillot, Paola Stefanelli, Marjolein van Gent, Guy Berbers, Didrik Vestrheim, Margrethe Greve-Isdahl, Lena Wehlin, Margaretha Ljungman, Norman K. Fry, Kevin Markey, Qiushui He

**Affiliations:** 1 Institute of Biomedicine, Department of Microbiology, Virology and Immunology, University of Turku, Turku, Finland; 2 Department of Pediatrics and Adolescent Medicine, Turku University Hospital, Turku, Finland; 3 Department of Microbiology, Universitair Ziekenhuis Brussel (UZ Brussel), Vrije Universiteit Brussel (VUB), Brussels, Belgium; 4 Statens Serum Institut, Infectious Disease Preparedness – Bacteria, Parasites and Fungi, Copenhagen, Denmark; 5 Department of Clinical Microbiology, Odense, University Hospital, Odense, Denmark; 6 Institut Pasteur, Centre National de Référence de la Coqueluche et autres Bordetelloses, Paris, France; 7 Department of Infectious Diseases, Istituto Superiore di Sanità, Rome, Italy; 8 Centre for Infectious Disease Control, National Institute for Public Health and the Environment (RIVM), Bilthoven, the Netherlands; 9 Department of Vaccine Preventable Diseases, Norwegian Institute of Public Health, Oslo, Norway; 10 Department of Microbiology, Public Health Agency of Sweden, Solna, Sweden; 11 Respiratory and Vaccine Preventable Bacteria Reference Unit, Public Health England – National Infection Service, London, United Kingdom; 12 National Institute for Biological Standards and Control, Potters Bar, United Kingdom; 13 Department of Medical Microbiology, Capital Medical University, Beijing, China

**Keywords:** pertussis, pertactin, acellular vaccine, vaccination, Europe

## Abstract

**Introduction:**

Pertussis outbreaks have occurred in several industrialised countries using acellular pertussis vaccines (ACVs) since the 1990s. High prevalence of pertactin (PRN)-deficient *Bordetella pertussis* isolates has been found in these countries.

**Aims:**

To evaluate in Europe: (i) whether proportions of PRN-deficient strains increased in consecutive collections of *B. pertussis* clinical isolates; (ii) if the frequency of PRN-deficient strains in countries correlated with the time since ACV introduction; (iii) the presence of pertussis toxin (PT)-, filamentous haemagglutinin (FHA)- or fimbriae (Fim)-deficient isolates.

**Methods:**

*B. pertussis* clinical isolates were obtained from different European countries during four periods (EUpert I–IV studies): 1998 to 2001 (n = 102), 2004 to 2005 (n = 154), 2007 to 2009 (n = 140) and 2012 to 2015 (n = 265). The isolates’ selection criteria remained unchanged in all periods. PRN, PT, FHA and Fim2 and Fim3 expression were assessed by ELISA.

**Results:**

In each period 1.0% (1/102), 1.9% (3/154), 6.4% (9/140) and 24.9% (66/265) of isolates were PRN-deficient. In EUpert IV, PRN-deficient isolates occurred in all countries sampled and in six countries their frequency was higher than in EUpert III (for Sweden and the United Kingdom, p < 0.0001 and p = 0.0155, respectively). Sweden and Italy which used ACVs since the mid 1990s had the highest frequencies (69%; 20/29 and 55%; 11/20, respectively) while Finland, where primary immunisations with ACV containing PRN dated from 2009 had the lowest (3.6%). Throughout the study, no PT- or FHA-deficient isolate and one Fim2/3-deficient was detected.

**Conclusion:**

Results suggest that the longer the period since the introduction of ACVs containing PRN, the higher the frequency of circulating PRN-deficient isolates.

## Introduction

Between 2010 and 2012, nationwide pertussis outbreaks occurred in many countries including Australia, the Netherlands, the United Kingdom (UK) and the United States (US). In all of these countries acellular pertussis vaccines (ACVs) had been introduced for booster or primary immunisations in the late 1990s or the early 2000s [1-4]. Currently used ACVs contain different combinations of pertussis toxin (PT), pertactin (PRN), filamentous haemagglutinin (FHA), and fimbriae 2 and 3 (Fim2/3) [5]. 

The genetic characteristics of *Bordetella pertussis* circulating strains have been investigated in relation to pertussis vaccine components [6]. It seems that after the introduction of ACVs, isolates not expressing the vaccine antigen PRN have appeared. *B. pertussis* PRN-deficient strains can cause typical symptoms of pertussis [7,8]. To date, isolates with these strains have been found in several countries, including Australia, Finland, France and the US [7-12]. PRN-deficient isolates have been increasingly reported and during the epidemics in 2010–12 the prevalence observed was high in the US (85%) and Australia (78%) [8,13]. Furthermore, since 2009, *B. pertussis* not expressing FHA nor PT and PRN was reported in France, Sweden and the US [14-16].

In Europe, vaccines and vaccination programmes vary in different countries [17]. To study genetic changes in *B. pertussis* populations in Europe, panels of *B. pertussis* isolates have been serially collected during four periods in a total of 11 European countries. The EUpert I panel was collected during 1999–2001 and included 102 isolates from five countries (Finland, France, Germany, the Netherlands and Sweden), the EUpert II in 2004–05 included 154 isolates from eight countries (Denmark, Finland, France, Germany, The Netherlands, Poland, Sweden and the UK), and the EUpert III in 2007–09 included 140 isolates from seven countries (Denmark, Finland, France, the Netherlands, Norway, Sweden and the UK). Four countries (Finland, France, the Netherlands, and Sweden) participated in all collections [12,18,19]. In the current study, the EUpert IV panel was collected from nine European countries: Belgium, Denmark, Finland, France, Italy, the Netherlands, Norway, Sweden, and the UK, during 2012–15 (time period was defined in the study contract). Altogether, 265 *B. pertussis* isolates were included. Selection criteria have remained unchanged for all four collections, which allows a unique opportunity to study and compare changes in bacterial populations over the past 15 years. Detailed vaccination programmes and vaccination coverage of the EUpert IV study countries are presented in Table 1 [2,4,9,17,20]. Vaccination programmes for the two countries (Germany, Poland) previously participating in EUpert studies have been published [19].

**Table 1:** Vaccination programmes with a focus on pertussis in nine European countries until 2015

Country	Start of vaccination against pertussis	Initial (primary)	Booster	PRN included	Approximate coverage (primary, %)
Belgium	1950s	2, 3, 4 mo DtwP	No booster	NA	NA
	1999 (Flanders)	2, 3, 4 mo DTaP-IPV-Hib	15 mo, 4–6 yr and 14–16 yr, DTaP-IPV-Hib	Yes	97–99
	2002 (Wallonia)	2, 3, 4 mo DTaP-IPV-Hib	15 mo, 4–6 yr and 14–16 yr, DTaP-IPV-Hib	Yes	97–99
Denmark	1961	5, 6, 7, 15 mo, DTwP	No booster	NA	80
	1969	5, 9 wk and 10 mo, wP	No booster	NA	80
	1997	3, 5, 12 mo, DTaP-IPV	No booster	No (solely PT)	85
	2002	3, 5, 12 mo, DTaP-IPV/Hib	No booster	No (solely PT)	85
	2003	3, 5, 12 mo, DTaP-IPV/Hib	5 yr, dTap	No (solely PT)	85/85^a^
	2004	3, 5, 12 mo, DTaP-IPV/Hib	5 yr, dTap-IPV	No (solely PT)	90/85^a^
Finland	1952	3, 4, 5 mo, DTwP	20–24 mo, DTwP	NA	95
	2003	3, 4, 5 mo, DTwP	20–24 mo, DTwP, and 6 yr, dtap	Yes (6 yr booster)	95–99
	2005	3, 4, 12 mo, DTaP-IPV-Hib	4 yr, DTaP-IPV-Hib, and 14 yr, dtap	No	95–99
	2009	3, 5, 12 mo, DTaP-IPV-Hib	4 yr, DTaP-IPV, 14–15 yr dtap	Yes	95–99
France	1959	3, 4, 5 mo, DTwP-IPV	16–18 mo, DTwP-IPV	NA	NA
	1995	2, 3, 4 mo, DTwP-IPV-Hib	16–18 mo, DTwP-IPV-Hib	NA	> 90/82^a^
	1998	2, 3, 4 mo, DTwP-IPV-Hib	16–18 mo, DTaP-IPV-Hib; 11–13 yr, DTaP-IPV	Yes (in boosters)	> 90/89^a^
	2004	2, 3, 4 mo, DTaP-IPV-Hib	16–18 mo, DTaP-IPV-Hib; 11–13 yr, DTaP-IPV; cocooning strategy, dTap-IPV	Yes/no (two vaccines)	> 90
	2008	2, 3, 4 mo, DTaP-IPV-Hib	16–18 mo, DTaP-IPV-Hib; 11–13 yr, DTaP-IPV; 26–27 yr, dTap-IPV cocooning strategy, dTap-IPV, for all adults who did not receive booster in last 10 yrs	Yes/no (two vaccines)	> 90
	2013	2, 4,11 mo, DTaP-IPV-Hib	6 yr, DTaP-IPV-Hib; 11–13 yr, dTap-IPV; 25 yr, dTap-IPV cocooning strategy, dTap-IPV, for all adults who did not receive booster in last 5 yrs	Yes/no (two vaccines)	> 90
Italy	1961	2, 4, 6 mo DTwP	No booster	NA	33
	1995	3, 5–6, 11–13 mo, DTaP-IPV-Hib	5–6 yr and 11–18 yr (10 yr after primary aP), DTaP-IPV	Yes	> 87
	2002	3, 5–6, 11–13 mo, DTaP-IPV-Hib	5–6 yr and 11–18 yr (10 yr after primary aP), DTaP-IPV	Yes	> 95
Netherlands	1953	3, 4, 5 mo, DTwP	4 yr, DTwP	NA	NA
	1962	3, 4, 5, 11 mo, DTwP-IPV	No booster	NA	NA
	1993	3, 4, 5, 11 mo, DTwP-IPV	No booster	NA	NA
	1999	2, 3, 4, 11 mo, DTwP-IPV	No booster	NA	97
	2001	2, 3, 4, 11 mo, DTwP-IPV	4 yr, DTaP-IPV	Yes (in booster)	97
	2003	2, 3, 4, 11 mo, DTwP-IPV-Hib	4 yr, DTaP	Yes (in booster)	97
	2005	2, 3, 4, 11 mo, DTaP-IPV-Hib	4 yr, DTaP-IPV	Yes	97
Norway	1952–1984	3, 4, 5, 15–18 mo, DTwP	No booster	NA	NA
	1984–1997	3, 5, 10 mo, DTwP	No booster	NA	NA
	1998	3, 5, 12 mo, DTaP	No booster	Yes	92
	2001	3, 5, 12 mo, DTaP-IPV + Hib	No booster	Yes	91
	2006	3, 5, 12 mo, DTaP-IPV or Hib	7 yr, DTaP-IPV	Yes	94
Sweden	1953	3, 5, 12 mo, DTwP	NA	NA	NA
	1979–1996	No vaccination against pertussis	NA	NA	NA
	1996	3, 5, 12 mo, DTaP	NA	Yes	98
	1998	3, 5, 12 mo, DTaP-IPV-Hib	NA	Yes	98
	2005	3, 5, 12 mo, DTaP-IPV-Hib	10 yr, DTaP, for those born 1996–2001	Not consistent, PRN has not been a component in all vaccines used, whereas PT and FHA have	98
	2007	3, 5, 12 mo, DTaP-IPV-Hib	5–6 yr, DTaP-IPV, for those born 2002; 14–16 yr, dTap	NA	98
United Kingdom	1957	DTwP completed by 6 mo	NA	NA	NA
	1968	3, 5, 11 mo, DTwP	NA	NA	NA
	1990	2, 3, 4 mo, DTwP	NA	NA	> 90
	2000–2001	2, 3, 4 mo, DTwP	4–6 yr, DTaP-Hib	Yes (in booster)	94
	2004	2, 3, 4 mo, DTaP-IPV-Hib	4–6 yr, DTaP-Hib	Yes	94
	2012	2, 3, 4 mo, DTap-IPV-Hib	3 yr 4 mo, DTaP-Hib + recommended maternal vaccination	Yes	94

DTaP: diphtheria-tetanus-acellular pertussis; dTap: diphtheria-tetanus-acellular pertussis (with reduced amount of diphtheria and pertussis antigens); dtap: diphtheria-tetanus-acellular pertussis (with reduced amount of diphtheria, tetanus and pertussis antigens); DTwP: diphtheria–tetanus–whole-cell pertussis; FHA: filamentous haemagglutinin; Hib: *Haemophilus influenzae* type b; IPV: inactivated poliovirus vaccine; mo: month; NA: not available; PRN: pertactin; PT: pertussis toxin; yr: year.

^a^ Primary/booster vaccination.

DTaP vaccines contain 1–3 pertussis components: (i) PT, (ii) PT and FHA or (iii) PT, FHA and PRN.

In this study, we aimed to evaluate whether there has been an increase in the proportion of PRN-deficient isolates among the clinical *B. pertussis* isolate collections in Europe over time. We also assessed if the proportions of PRN-deficient isolates detected in given study countries in 2012–15 were related to the time since these countries had introduced ACVs containing PRN. Furthermore, the presence of PT-, FHA- or Fim-deficient isolates in all collected isolates was investigated.

## Methods

### Study context and isolate panels

The total material comprised 661 *B. pertussis* isolates, which were collected during the four study periods (EUpert I–IV) from 1998 to 2015. The genotyping results of EUpert I–III studies have been previously published [12,18,19]. In addition, data relating to the expression (serotyping) of Fim2/3 for EUpert I–III collections have also been published (no Fim2/3-deficient isolates found), as well as PRN expression data of the EUpert III collection, showing PRN-negative isolates in France, Norway and Sweden [18,19]. 

In this study, the data from EUpert I–III were completed, by screening for PRN expression in EUpert I and II panels (n = 256). Moreover, FHA expression in EUpert I, II and III panels (n = 396) was tested (country-based data not shown). 

The study also included the full analysis of EUpert IV isolates (n = 265 isolates), which were investigated for the presence of PT, FHA, PRN and Fim2 and Fim3. 

### Selection criteria for isolates and collection of patient data

The recommended number of isolates was 30 per country and the selection criteria for isolates in the EUpert IV study were the same as those used in the previous EUpert I–III studies [19]. 

These were as follows:

(i) *B. pertussis* isolates should be selected from different geographical regions in a given country and be epidemiologically unrelated.

(ii) An equal number of isolates from vaccinated (n = 15) and unvaccinated individuals (n = 15) should be collected. Isolates are preferred to be selected from individuals younger than 5 years of age.

(iii) For those countries with large numbers of isolates in their collections, isolates should be randomly selected in addition to the above criteria.

The following numbers of isolates were received per country: Belgium (n = 38), Denmark (n = 27), Finland (n = 28), France (n = 29), Italy (n = 20), the Netherlands (n = 32), Norway (n = 32), Sweden (n = 29), and the UK (n = 30). For Italy, all isolates were collected from the Rome area as no other isolates were available. 

Data collection included original code of isolate, country, date of collection, city and available characteristics of patients. The latter included sex, age, vaccination status, number of doses received and hospitalisations due to pertussis.

### Culture

*B. pertussis* isolates were shipped in frozen storage tubes to the University of Turku (UTU), Finland. Isolates were cultured on Regan-Lowe medium (without cephalexin) at + 35 °C for 48h to determine production of PRN, FHA, Fim2 and Fim3. To determine production of PT, isolates were cultured at + 35 °C with 5% CO_2_ for 48h [21]. The subculture of each isolate was kept minimal, although the exact number of subculture is not known.

### Detection of antigen expression

Detection of PRN, PT, FHA and Fim2 and Fim3 expression was performed by specific enzyme-linked immunosorbent assay (ELISA) as described previously [7,21,22]. In the ELISA, whole bacterial cells were used as coating antigen. Production of different antigens (PT, FHA, PRN, Fim2 and Fim3) was detected with specific monoclonal antibodies, kindly provided by the National Institute for Biological Standards and Control (NIBSC) in the UK and the National Institute for Public Health and the Environment (RIVM) in the Netherlands. Three French strains including FR3496 (negative for PT), FR3693 (negative for PRN), and FR4624 (negative for FHA), as well as two Swedish Fim2- or Fim3-expressing strains S1 (Fim2) and S3 (Fim3) were used as controls.

### Sequencing of the whole *prn* gene

To investigate the mechanisms for PRN-deficiency of the EUpert IV collection, the *prn* gene was first amplified and sequenced afterwards. Only isolates with detected PRN-deficiency were sequenced. All primers are presented in Table 2. The method and some of the primers have been described previously [12,23]. Each reaction mixture was preheated for 5 min at 95 °C. Thirty amplification cycles were used with following programme: 95 °C for 30s, 59 °C for 30s and 90s at 72 °C followed by a final cycle at 72 °C for 7 min. Sequencing was performed at the Institute for Molecular Medicine Finland (FIMM) laboratory (Helsinki, Finland).

**Table 2:** Primers used in this study for PCR amplification and sequencing

Name of primer	Sequence (5’–3')	Target (position on the genome)^a^
PrnG_A2 for	CCC ATT CTT CCC TGT TCC AT	*prn* gene amplification & sequencing, first part (1098027–1098046)
PrnG_A rev	TGT TGG CAA GGG TAA AGG TC	*prn* gene amplification, first part (1099698–1099717)
Prn-BF_1322bp	AGC TGG GCG GTT CAA GGT	*prn* gene amplification & sequencing, second part (1099488–1099505)
PrnGeneR	GCC TGA GCC TGG AGA CTG G	*prn* gene amplification, second part (1100877–1100895)
PrnProm FOR	GCT CAA AGC AGG AAA AAG CA	*prn* promoter amplification & sequencing (1097635–1097654)
PrnProm REW	CGCTTACCTTGATGGTGGTT	*prn* promoter amplification (1098288–1098307)
PrnSeq1F	GCC AAT GTC ACG GTC CAA	*prn* gene amplification, sequencing (1098595–1098612)
PrnSeq2F	TGT CGA TCA CCT TGC AGG	*prn* gene amplification, sequencing (1099166–1099183)
PrnSeq3F	AAC GGC AAT GGG CAG TG	*prn* gene amplification, sequencing (1099765–1099781)
PrnSeq4F	GACAGCGGTTTCTACCTGGA	*prn* gene amplification, sequencing (1100269–1100288)

^a^ Position based on the genome of Tohama I (GenBank accession number: NC 002929).

### Statistical analysis

Fisher’s exact and chi-squared tests to assess differences between vaccinated and unvaccinated patients and frequency of PRN-deficient isolates between EUpert I-IV collections were calculated using GraphPad prism 4·0 version (San Diego, California (CA), US). Two-tailed p values < 0·05 were considered significant. To analyse the trend in PRN-deficiency over time, the proportions of PRN-deficient isolates per country were estimated using logistic regression with the year of diphtheria-tetanus-acellular pertussis (DTaP, containing PRN) implementation in the country as the independent variable. Results were given as odds ratios (OR) between start of ACV and frequency of PRN-deficient isolates. The analysis was conducted using the R software (R foundation).

## Results

### Pertactin expression

**Figure 1:**
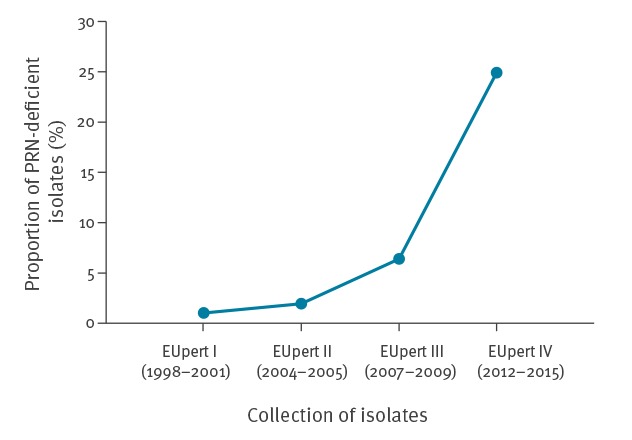
Proportion of pertactin-deficient isolates in EUpert I–IV collections, Europe, 1998–2015 (n = 661 isolates)

In EUpert I, one (1.0%; 1/102; Sweden (n=1)) PRN-deficient isolate was found. In EUpert II three (1.9%; 3/154; France (n=1), Germany (n=1), the Netherlands (n=1)) and in EUpert III nine (6.4%; 9/140; France (n=5), Norway (n=3), Sweden (n=1)) such isolates were detected. In EUpert IV, 66 (24.9%; 66/265) isolates were deficient for PRN (Figure 1). Significant increase of PRN-deficient isolates was found in EUpert IV compared with the three previous EUpert studies (p < 0.0001). Furthermore, PRN-deficient isolates were found in all nine study countries in different proportions as shown in Table 3. In five countries (Denmark, Finland, France, the Netherlands and Norway) frequency of PRN-deficient isolates seemed to have increased when compared to EUpert III, moreover in two (Sweden and the United Kingdom) the increase was significant (p < 0.0001 and p = 0.0155, respectively). Among the PRN-deficient isolates, 56 (84.9%) were Fim3, nine (13.6%) Fim2 and one (1.5%) Fim2,3 serotype. 

PRN: pertactin.

The number of isolates tested in the four periods was 102 in EUpert I (1998–2001), 154 in EUpert II (2004–2005), 140 in EUpert III (2007–2009) and 265 in EUpert IV (2012–2015) from 11 countries with distinct vaccines and vaccination strategies.

**Table 3:** Number of pertactin-deficient *Bordetella pertussis* isolates in study countries in the EUpert IV collection, Europe, 2012–2015 (n = 66 isolates)

Country	Number of PRN-deficient isolates in EUpert IV	Number of isolates collected in EUpert IV	Frequency of PRN-deficient isolates (%) in EUpert IV (95% CI)	Number of PRN-deficient isolates in EUpert III	Number of isolates collected in EUpert III	Frequency of PRN-deficient isolates (%) in EUpert III (95% CI)	EUpert IV vs. EUpert III (p value)	Year of ACV introduction for primary vaccination^a^
Sweden	20	29	69.0 (50.8–82.7)	1	19	5.0 (0.9–23.6)	<0.001 (increase)	1996
Italy	11	20	55.0 (34.2–74.2)	NA	NA	NA	NA	1995
Norway	10	32	31.3 (18.0–48.6)	5	20	25.0 (11.2–46.9)	0.7574	1998
United Kingdom	8	30	26.7 (14.2–44.5)	0	20	0.0 (0.0–16.1)	0.0155 (increase)	2004
France	5	29	17.2 (7.6–34.6)	3	20	15.0 (5.2–36.0)	1.0000	2004
Denmark	4	27	14.8 (5.9–32.5)	0	23	0 (0.0–14.3)	0.1147	1997^b^
Belgium	4	38	10.5 (4.2–24.1)	NA	NA	NA	NA	1999 (Flanders) / 2002 (Wallonia)
Netherlands	3	32	9.4 (3.2–24.2)	0	20	0 (0.0–16.1)	0.2760	2005
Finland	1	28	3.6 (0.6–17.7)	0	16	0 (0.0–19.4)	1.0000	2005^c^

ACV: acellular pertussis vaccine; CI; confidence interval; FHA: filamentous haemagglutinin; NA: not available; PRN: pertactin; PT: pertussis toxin.

^a^ The years in this column are given for the introduction of a PRN-containing ACV except if otherwise stated.

^b^ In Denmark, solely PT-based vaccine in use from 1997.

^c^ In Finland, vaccine containing PT and FHA was first used, and PRN was included in 2009.

**Figure 2:**
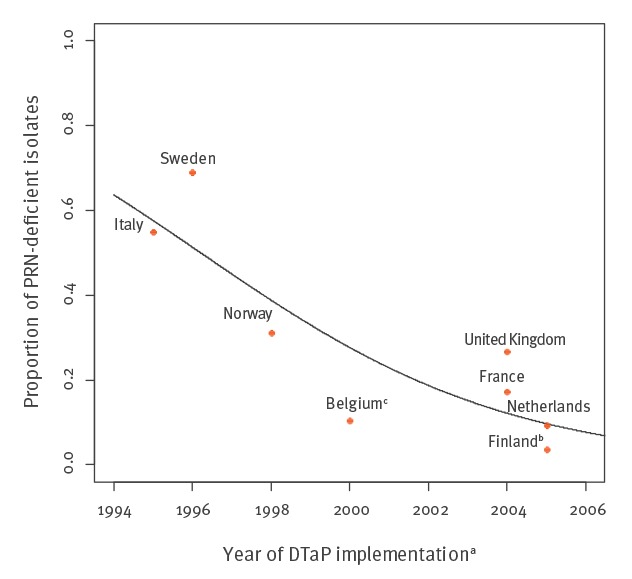
Correlation between the introduction of a primary acellular pertussis vaccine containing pertactin (PRN) in a European country and the proportion of PRN-deficient isolates found in the study, 2012–2015

ACV: acellular pertussis vaccine; DTaP: diphtheria-tetanus-acellular pertussis; FHA: filamentous haemagglutinin; PRN: pertactin; PT: pertussis toxin.

^a^ Year when an ACV containing PRN was implemented for primary vaccination, except for Finland where the year of introduction of ACV not containing PRN is shown (see footnote b).

^b^ In Finland, ACV containing PT and FHA was first used in 2005 and ACV containing PRN was introduced in 2009.

^c^ In Belgium, ACV was introduced in 1999 in Flanders (representing 60% of the population) and in 2002 in Wallonia (representing 40% of the population); therefore, a balanced mean of introducing year (2000.2y) was used in calculations.

The distribution of the PRN-deficient isolates and their frequencies in the EUpert IV-study countries is shown in Table 3 together with the year of introduction of ACVs. The highest frequencies of PRN-deficient isolates were found in Sweden (69.0%), Italy (55.0%), Norway (31.3%) and the UK (26.7%). The lowest numbers were found from Finland and the Netherlands (< 10% in both). The difference in frequencies between Sweden, Italy, Norway and the UK versus Finland were all significant (all p < 0.05), whereas for the Netherlands, a significant difference was only observed with Sweden and Italy (both p < 0.001). Figure 2 shows the proportion of PRN-deficient isolates observed in EUpert IV study and the implementation year of primary DTaP immunisation in all countries, except Denmark where DTaP vaccine containing only one pertussis antigen, PT, is used. Denmark is the only country in Europe using this vaccine. The correlation between year of introduction of ACV and PRN-deficient isolates in Denmark was however checked, and was lower than for most of the other countries (data not shown). For the other countries, an OR of 1.29 (95% CI: 1.18–1.41) was found, meaning that every year after the introduction of DTaP vaccination the odds to have PRN-deficient strains increases with 29%.

Denmark is not included in the figure, as a monocomponent PT vaccine is used in this country since 1997.

### Molecular mechanisms causing pertactin deficiency

The entire *prn* gene was sequenced. We found six different mechanisms causing PRN expression to be suppressed (Table 4). Of the 66 PRN-deficient isolates, 31 (47.0%) had the insertion of the *IS481* element at position 1613^1614 (from the beginning of the *prn* gene) either in forward or reverse orientation and one had it at position 2735^2736. *IS481* element is found in multiple copies in the genome of *B. pertussis* and helps it to adapt to different immunised populations [24]. The second common cause (18 isolates; 27.3%) was a large inversion in the *prn* promoter area. In one isolate, a nucleotide mutation of a G to a T at position 2077 leading to a stop codon, which was not previously described, was found. Other causes were single point mutation and deletion, and deletion of the entire *prn* gene. In addition, the mechanism behind PRN-deficiency for one isolate was not located within the *prn* gene, although repeated sequencing was performed.

**Table 4:** Mechanisms causing pertactin deficiency in *Bordetella* *pertussis* isolates of the EUpert IV collection, Europe, 2012–2015 (n = 66 isolates)

Mechanism	Country and number of isolates with a given mutation									Total	%
	Belgium	Denmark	Finland	France	Italy	Netherlands	Norway	Sweden	United Kingdom		
Insertion of *IS*481 element	2	2	0	0	8	1	3	11	5	32	48.5
Inversion of 22kb of *prn* promoter	2	0	0	5	1	2	0	7	1	18	27.3
223STOP: C > T	0	0	0	0	0	0	6	1	1	8	12.1
631^632STOP: ΔT	0	1	0	0	2	0	0	1	0	4	6.1
Δ*prn*	0	1	0	0	0	0	1	0	0	2	3.0
2077STOP: G > T	0	0	1	0	0	0	0	0	0	1	1.5
Unknown	0	0	0	0	0	0	0	0	1	1	1.5
Total	4	4	1	5	11	3	10	20	8	66	100.0

Δ: deletion; >: mutation; ^: position of the insertion in the gene; *prn*: pertactin gene; STOP: stop codon in the gene.

### Pertactin deficiency, vaccination status and patient data

Of 265 patients in the EUpert IV study, 130 (49.1%) were vaccinated and 135 (50.9%) unvaccinated. PRN-deficient isolates were isolated from 66 patients. Of them, 16 (24.2%) were partially vaccinated, 10 (15.2%) had received full primary vaccination, six (9.1%) had full primary and booster vaccination, 32 (48.5%) were unvaccinated and for two patients (3.0%) the vaccination status was unknown. No significant difference in proportion of PRN-deficient isolates was found between vaccinated and unvaccinated patients among study countries, except in the UK where these isolates were found more in vaccinated patients (p = 0.0348). PRN-deficient isolates were found mostly from infants less than one year of age (39/66; 59.1%), but the age ranged from < 1 month to 55 years. Hospitalisation data were available only for 41 patients and showed a range from 0 to 28 days. Complications data were only available for 11 patients with one having thrombocytopenia and one bradycardia and hypoxia, for the rest no severe complications were notified. From these patients, only one was vaccinated.

### Pertussis toxin, filamentous haemagglutinin and fimbrial expression

Among isolates collected during the four studies (n=661), one (0.15%) did not produce Fim2 or Fim3. This isolate was found in Norway and originated from the EUpert IV collection. No difference in bacterial growth and colony formation on charcoal agar was noticed between Fim-positive and -negative isolates. In the EUpert IV study, 120 (45.3%), 141 (53.2%) and three (1.1%) isolates carried Fim2, Fim3 and Fim2,3 serotypes, respectively. No other Fim-deficient isolate was found.

No FHA-deficient isolates were found among EUpert I–IV collections. No PT-deficient isolates were found among the EUpert IV collection.

## Discussion

In this study, serial collections of *B. pertussis* isolates were investigated from 1998 to 2015 in European countries where different vaccines and vaccination strategies are used. The countries submitting isolates varied between collections, however Finland, France, the Netherlands and Sweden contributed to all of them [12,18,19]. In the latest collection, nine countries were included. The frequency of *B. pertussis* PRN-deficient isolates was low (13/396; 3.2%) during the period 1998–2009 but this had increased in 2012–2015 with a prevalence of 24.9% (Figure 1). Our findings are in line with previous studies from the US and Australia, where the prevalence of PRN-deficient isolates has increased significantly during 2010-2012 [13,14]. 

The coverage for primary vaccination has been high since the introduction of ACVs in all study countries (> 90%), suggesting that there is a selective pressure for *B. pertussis* to inactivate the PRN-production. It is known that population density plays a role in transmission of bacterial pathogens. However, this does not seem to affect increased circulation of PRN-deficient isolates, because, although the Netherlands has a high population density, the frequency of PRN-deficient isolates has only increased slightly during the past 10 years. On the other hand both Sweden and Finland have low population densities, but significantly different frequencies of PRN-deficient isolates.

All study countries in the two last EUpert collections have implemented either ACV booster vaccination or changed to ACV primary vaccination in the 1990s or at the beginning of 2000 (Tables 1 and 3) [2,17,20]. Therefore, we made a comparison between the introduction of ACV and the increase in PRN-deficient isolates. As shown in Figure 2, it appears that countries which introduced primary ACV vaccination earliest have a higher proportion PRN-deficient isolates in 2015 than those where primary ACV vaccination was introduced more recently. Sweden and Italy were the first two countries where ACVs were introduced in Europe and the proportions of PRN-deficient isolates in these countries (69.0% and 55.0%) are significantly higher than other countries (Table 3). If we compare Finland and the Netherlands, both switched to ACV in 2005, but Finland used first ACV containing PT and FHA and started to use PRN in the three-component vaccine from 2009 (Table 1). This is reflected in the frequency of PRN-deficient isolates observed in the Netherlands (9.4%) and in Finland (3.6%). Our study supports the hypothesis that the emergence of PRN-deficient isolates is dependent on the timing since the introduction of ACV containing PRN.

In Europe, Poland is the only country using whole cell vaccines for primary vaccination. Unfortunately Polish isolates were not available in the latest EUpert III and IV studies. In EUpert II study, 13 isolates were available [25]. None was found to be PRN-deficient.

Our results showed that PRN-deficient isolates were also found in Denmark, where a monocomponent PT vaccine has been used more than 15 years [26]. This finding is very interesting as without PRN in the vaccine, there should not be a vaccine derived selection for PRN-deficient *B. pertussis* isolates. One possible explanation might be that the patients in Denmark were infected with PRN-deficient strains from other neighbouring countries like Sweden and Norway where the frequency of PRN-deficient isolates is high. Indeed, the four PRN-deficient isolates observed in Denmark were caused by three different mechanisms (Table 4), which also mediated PRN-deficiency in other countries. In addition, PRN-deficient isolates have shown to have positive advantage in causing disease in vaccinated population (diphtheria–tetanus–whole-cell pertussis or DTaP) [8]. A recent study in mice demonstrated that a PRN-deficient isolate colonised the respiratory tract more effectively than PRN-positive isolate in ACV-immunised mice [27]. It is possible that these isolates could have similar advantages in colonisation and disease development in human populations. Travel/commute information could be collected in the future to show the possible effect on strain shifting from one country to another. 

The correlation between year of introduction of ACV and PRN-deficient isolates was however lower for Denmark than for most of the other countries, except Belgium where PRN-containing ACV was introduced in Flanders and Wallonia in 1999 and 2002, respectively. According to Pierard (personal communication April, 2017) ca 60% of Belgian children (less than 17 years of age) received ACV in 1999 and 40% in 2002. This may explain the low frequency of PRN-deficient isolates found in Belgium in this study. 

Previously, it has been reported that PRN-deficient isolates are more common in vaccinated than in unvaccinated individuals [8]. In this study we did not find such a difference. When we compared the vaccination data of patients with PRN-deficient isolates, the distribution between vaccinated and unvaccinated individuals was close to 1:1 (excluding the UK). This may suggest that PRN-deficient strains might be able to infect individuals regardless of vaccination status. This could also partly explain why the PRN-deficient isolates were found in Denmark. However, a further prospective study in which detailed clinical information of patients is collected would be needed to explore these hypotheses. It is also worth keeping in mind that the number of isolates isolated from vaccinated and unvaccinated individuals included in the present study was very limited and there might be a selection bias because of the high number of infants in our study. 

In this study, seven different molecular mechanisms were found to mediate PRN deficiency for *B. pertussis*. Five mechanisms have been reported previously [9,12,28]. A new deletion of thymine in position 631^632 (4 isolates) and point mutation of G > T in position 2077 (1 isolate) was found. In addition, the mechanism for one isolate was not located within the *prn* gene.

The appearance of PRN-deficient isolates was first described in Italy and France with only a few isolates in 2007 [9,11]. During the period of 2000–10, frequencies of PRN-deficient isolates started to increase and reached 5–8% prevalence in France, Australia and the US. ACVs were introduced in Australia and in the US in the late 1990s [13,16,28]. However, after 2010 the prevalence of PRN-deficient isolates had increased dramatically in Australia (78%) and the US (85%), whereas in France it increased slightly to 15% [8,13,29]. In Japan ACV vaccination has been used since 1981 and the first detection of PRN-deficient isolates was reported in 2012 on isolates from 1995–1999. During the period 1990–1994 only eight (5%) such isolates were detected, whereas the prevalence increased to 32–38% in 2000–2009 [30]. However, a recent report from Japan showed that frequency of PRN-deficient isolates decreased to 8% in 2014–2016. The authors reported that vaccines not including PRN have been introduced since 2012 and may have resulted in the low prevalence [31]. Results from all these studies are in line with our findings and suggest that number of PRN-deficient *B. pertussis* isolates is increasing constantly 10–15 years after the introduction of ACV vaccination containing PRN.

In this study we did not find any PT-deficient isolates, even from Denmark, although the selective pressure from the monocomponent PT vaccine is considered to be high. Moreover, only a few PT-deficient isolates have been reported previously [9,14,16]. This confirms that PT is important for *B. pertussis* and plays an essential role in pathogenesis of pertussis. However, the number of isolates from Denmark was limited so we cannot conclude that PT-deficient isolates are not present in Denmark. We did not detect any FHA-deficient isolates either. As FHA is dominant in adhesion and is needed for tracheal colonisation, it seems to be conserved in *B. pertussis* [5]. For fimbriae, only one isolate that did not express Fim2 or Fim3 was isolated in Norway in 2013. Previously, one study from Japan described a similar finding among Japanese isolates [32]. However, shifts from Fim2 to Fim3 or vice versa may be common among circulating isolates after epidemic seasons, so it is difficult to predict how the expression will change and whether the appearance of Fim2/3-deficient isolates will increase or not.

There are both strengths and limitations in this study. The strengths of this study are that (i) we have a serial collection of isolates during the past 15 years, (ii) selection criteria have been the same for all collections, (iii) we have isolates from countries with different vaccination programmes and therefore, the impact of ACV introduction could be assessed, (iv) the place of origin is known for all isolates and shows that these isolates were not collected from local outbreaks and (v) all analyses were done by one laboratory with the same protocol so the results are comparable. The limitations included are as follows: (i) clinical information, including e.g. length of hospitalisation and severe complications were not available from many study patients (159 of 265), (ii) the number of isolates included from each country was very limited and not proportional to population size. However, they do comprise almost all isolates available in many countries such as Denmark, Finland and Italy, where the use of culture for diagnosis of pertussis is diminishing. For those countries with limited number of isolates, it would be beneficial if DNA from clinical specimens could be used directly for detection of PRN-deficient isolates e.g. through PCR and sequencing. (iii) The epidemiological pressure of pertussis varies in European countries which could have an effect on the spread of new emerging strains, like in Finland the incidence was continuously decreasing and was very low during the period of EUpert IV study [33].

In conclusion, the prevalence of PRN-deficient *B. pertussis* isolates has increased in Europe and the increase seems to be associated with the time since the introduction of ACVs. PRN-deficient isolates were found in all nine study countries during the latest collection and the isolates were isolated from both vaccinated and unvaccinated individuals. The highest number of these isolates was found in Sweden and Italy, which were the earliest adopters of ACV among the study countries. Our study also underscores the importance of long-term surveillance of *B. pertussis* antigen production because of its possible impact on the effectiveness of vaccines.
